# Study on spatial accessibility and supply-demand fit of public older adult care facilities in Guangzhou

**DOI:** 10.3389/fpubh.2026.1915638

**Published:** 2026-09-18

**Authors:** Xiu Chen, Lianhua Liu, Jiaxin Luo, Wentao Tan, Luocong Qi

**Affiliations:** 1 Guangzhou Huashang College, Guangzhou, China; 2 Faculty of Humanities and Social Sciences, Macao Polytechnic University, Macau, China

**Keywords:** Guangzhou, public older adult care service facilities, spatial accessibility, supply–demand matching, walking accessibility

## Abstract

Against the backdrop of deepening aging population phenomenon, the coordination between the allocation of older adult care services and the needs of the older adult population has become a critical issue for optimizing urban public services. Taking the 11 administrative districts in Guangzhou as the research subjects, this study employs the nearest neighbor index, kernel density analysis, standard deviation ellipse, network accessibility analysis, and supply–demand matching models to explore the spatial distribution, accessibility, and supply–demand matching characteristics of public older adult care service facilities. The results indicate that both the demand and supply of older adult care services in Guangzhou exhibit significant spatial clustering, with the central urban area forming a core cluster of service demand and resource allocation. While the overall accessibility of public older adult care facilities is relatively high, it follows a spatial pattern of higher concentration in the center and lower in the periphery. The study suggests that the allocation of older adult care service resources in Guangzhou is generally reasonable, but regional disparities persist. Future efforts should further optimize facility layouts to enhance the balance and accessibility of older adult care service resource allocations.

## 1 Introduction

Population aging is a global social trend, and the deepening wave of aging has profoundly reshaped urban demographic structures and posed new demands on public services, social security, and spatial planning. By the end of 2024, China’s population aged 60 and above had surpassed 300 million. Against the backdrop of accelerating aging, traditional family-based older adult care models are under pressure, while the demand for socialized older adult care continues to rise. Properly planning and allocating public older adult care facilities, improving service accessibility, and achieving precise matching of resources and needs have become core tasks for promoting active aging and enhancing urban public service systems.

As a national central city and the core engine of the Guangdong-Hong Kong-Macao Greater Bay Area, Guangzhou boasts a high urbanization level and a large population and has now entered a stage of deep aging. According to the Guangzhou Statistical Yearbook (2025), as of the end of 2024, the permanent population of Guangzhou was approximately 18.97 million, with the aging population trend intensifying. The proportion of people aged 60 and above accounted for 19.81% of the permanent population, marking the city’s entry into a deep aging stage. The spatial distribution of the older adult population exhibits distinct characteristics, with high concentration in the central urban areas and a more scattered distribution in peripheral districts. There are significant differences in older adult care service demands across different regions. As the aging and high-age population trends intensify, the demand for public older adult care facilities such as community-based care, medical and nursing services, and rehabilitation continues to grow, placing higher requirements on the scientific layout, spatial accessibility, and supply–demand alignment of older adult care service facilities. In recent years, Guangzhou has continuously improved its integrated home-community-institutional older adult care service system, expanded various public older adult care facilities, and steadily enhanced service supply capacity. However, due to factors such as terrain, road networks, urban development patterns, and population mobility, the allocation of regional older adult care resources still faces significant contradictions: central urban areas have dense facilities and service loads nearing their limits, while remote areas like northern Conghua and northeastern Zengcheng exhibit significant facility shortages. There are both supply gaps and idle facilities in some areas, which restricts the equal development of older adult care services. Therefore, taking Guangzhou as the research object, analyzing the spatial accessibility and supply–demand adaptation characteristics of public older adult care service facilities not only helps to reveal the problems in the spatial allocation of older adult care resources, but also provides scientific basis for optimizing the layout of older adult care service facilities, promoting balanced development of older adult care services, and actively responding to population aging.

This article takes 11 administrative districts in Guangzhou as the research scope, using nearest neighbor index, kernel density analysis, standard deviation ellipse, network accessibility, and supply–demand adaptation models to systematically analyze the spatial pattern, pedestrian accessibility level, and supply–demand relationship of public older adult care service facilities, and to analyze service shortcomings and existing problems. Compared with existing research, which focuses on the spatial distribution or accessibility evaluation of older adult care service facilities, this paper constructs an integrated analysis framework of “spatial distribution characteristics spatial accessibility supply–demand adaptability,” combining spatial distribution analysis, network accessibility evaluation, and supply–demand adaptability analysis to comprehensively evaluate the level of public older adult care service resource allocation from the three dimensions of demand, supply, and spatial accessibility. Based on empirical results, differentiated optimization strategies are proposed to provide scientific basis for the balanced and high-quality development of older adult care services in Guangzhou, and to provide reference for the planning and resource optimization of public older adult care service facilities in similar mega cities in China.

## 2 Literature review

### 2.1 Research on the spatial layout and agglomeration characteristics of older adult care service facilities

The spatial distribution pattern of older adult care service facilities is the foundation of resource allocation. The academic community generally uses POI big data and demographic data as the basis, combined with GIS spatial analysis methods such as nearest neighbor index, kernel density analysis, standard deviation ellipse, Ripley K function, etc., to analyze the agglomeration, dispersion, and distribution trends of facilities, and study scales covering multiple levels such as provinces, urban agglomerations, mega cities, districts, and communities (1, 2). From the common pattern of spatial form, the majority of older adult care service facilities in domestic cities exhibit a circular distribution characteristic of core agglomeration and sparse periphery. The central urban area of the city has become a high-density gathering place for various older adult care facilities due to its high population density, complete supporting facilities, and profound development background; However, the layout of facilities in suburban, remote, and county-level areas is scattered and low-density, and the phenomenon of differentiation between urban and rural areas and urban areas is very prominent (3–5). Research on megacities represented by Shanghai and Beijing has revealed the evolutionary characteristics of their facility spatial patterns (6, 7). In Shanghai, the older adult-care facility pattern has shifted from early single-core clustering to a multi-center structure coupled with residential and transportation functions. In contrast, Beijing exhibits spatial mismatch between facility clusters and older adult population migration-older adult care beds remain concentrated in the central districts while the aging population relocates outward, causing increased resource pressure in peripheral areas. Comparing the evolutionary paths of two cities reveals two types of differentiated development paradigms. Existing results only independently describe the cross-sectional pattern of a single city, without comparing the adaptability differences between the two layout modes from the perspective of supply and demand coupling, nor establishing a multi spatial tool linkage framework to analyze the linkage mechanism between population migration and facility layout. Single dimensional analysis is difficult to reveal the inherent causes of the differentiation in older adult care resource allocation under the background of urban expansion.

There are significant differences in the layout logic of different types of older adult care facilities. Community older adult care and meal assistance embedded facilities tend to be closer to residential communities and highly overlap with the distribution of the older adult population; Large older adult care institutions, on the other hand, balance land use conditions with medical facilities, and some cities have formed new facility gathering points in the suburbs. In terms of factors affecting facility layout, existing research has formed a unified understanding that the size and spatial distribution of the older adult population are the core factors determining facility location, while transportation accessibility, distribution of medical resources, regional economic level, and land supply conditions also significantly affect facility location layout (3), (8). Gu Hongliang et al. (1) and Feng Huicheng et al. (9) confirm through decision-tree and machine-learning simulations that the planning of older adult care facilities should integrate surrounding public service attributes, with population concentration remaining the primary determinant. Methodologically, while traditional spatial statistics still prevail, big-data analytics and machine-learning algorithms are increasingly employed for scenario simulations, indicating a methodological transition toward data-driven planning (5). Traditional spatial statistics and machine learning have their own applicable boundaries. The former can only statically identify point clusters, while the latter often ignores the constraints of older adult walking; Existing research generally uses a single type of tool for cross-sectional analysis, lacking a comprehensive analysis approach that couples multiple methods, and unable to simultaneously characterize the matching degree between facility agglomeration forms and population needs. Although there have been abundant spatial characterization results in related research, most studies only rely on a single spatial statistical tool to complete pattern description, and there is insufficient exploration of the underlying mechanisms behind facility agglomeration. It is also difficult to combine the spatial organizational characteristics of facilities with the development needs of population aging for comprehensive efficiency analysis.

### 2.2 Research on spatial accessibility of older adult care service facilities

Spatial accessibility is a key dimension of evaluating convenience and equity in older adult care resource distribution. Considering walking as the primary travel mode for older adult groups, recent studies emphasize network-based walking accessibility rather than abstract Euclidean distance (10, 11). The 2-Step Floating Catchment Area (2SFCA) method and its subsequent variants—Gaussian, multi-modal, and layered 2SFCA—dominate evaluation frameworks (10, 12). There are obvious differences in distance attenuation and crowd threshold setting among the basic, Gaussian, and stratified 2SFCA models. Existing empirical studies mostly select a single model for cross-sectional measurement, lacking parallel verification and comparison of multiple models, making it difficult to verify the robustness of the measurement results. At the same time, there are few studies that embed differentiated walking tolerance features of older adult and young people into accessibility evaluation systems. Research on accessibility has evolved from static measurements to multimodal integration. Models incorporating distance attenuation parameters and differential travel distance thresholds enhance realism, while studies grounded in social stratification theory examine accessibility inequalities across income and age groups (13). Furthermore, integration with machine learning and spatial-regression models has enhanced the accuracy and flexibility of accessibility modeling (14, 15).

Based on the research conclusions of spatial differentiation in accessibility, the pattern of high center and low periphery is commonly found in cities across the country. Gong Peixu et al. (2026) pointed out that some high-density megacities have differentiated characteristics: Shanghai center’s central urban area has a high concentration of population, while basic older adult care facilities such as catering have a hidden weakness of insufficient accessibility, breaking the single cognition of optimal accessibility in the central area (14). The 15 min community living circle has become a hot topic in accessibility research. Chang et al. (15) divided service levels around the 5/10/15 min walking circle and evaluated the coverage gap of older adult care facilities within the living circle. The central urban area has a dense road network and sufficient facilities, with a high coverage rate within a 15 min walk; However, urban edges, mountainous areas, and suburban villages and towns are limited by terrain, road networks, and facility density, resulting in a significant increase in walking time and becoming service weak areas (16, 17). Moreover, Wei et al. (18) reveal a “false fairness” phenomenon whereby high motor-vehicle accessibility conceals the lack of walkable services for older adult residents, highlighting the need to integrate behavioral accessibility into spatial equity research. The existing accessibility research separates the evaluation systems of motor vehicles and walking, and the two sets of measurement standards cannot be compared with each other. Moreover, most studies only measure the physical distance, without refining the evaluation of service equalization based on the weight of the needs of older adult people with different mobility abilities. A single perspective is difficult to fully reflect the real differences in service acquisition among the older adult population. Although accessibility assessment methods have undergone continuous iteration and updates, most existing research focuses primarily on the physical distance between facilities and residential areas, lacking in-depth analysis of actual service delivery effectiveness and functional equity. There remains limited empirical exploration into how spatial accessibility disparities translate into inequities in older adult care services, or how facility layouts can be optimized to align with these spatial characteristics.

### 2.3 Research on the matching of supply and demand of older adult care services and equity

On the basis of research on spatial layout and accessibility, the academic community has further turned to the coupling relationship between supply and demand and spatial equity research, aiming to determine whether the allocation of older adult care resources is compatible with the needs of aging population. This field mainly uses coupling coordination models, supply–demand matching models, Gini coefficient, Lorenz curve, spatial autocorrelation and other methods to analyze from three dimensions: coordination degree, scale matching, and spatial equity. It is also the core research focus of the current older adult care planning field (12, 19).

In terms of supply and demand adaptation evaluation, the coupling coordination degree model is the most widely used research tool, often used to measure the overall synergy level between the older adult service supply system and the older adult demand system. Existing research shows that there is a significant spatial gradient difference in the supply and demand of older adult care services in Chinese cities: traditional central urban areas have a high degree of aging, resource aggregation, and generally high supply–demand coordination; The aging pressure in peripheral urban areas, counties, and rural areas is gradually increasing, but the follow-up of older adult care supply is insufficient, and the problem of supply–demand imbalance is prominent (19, 20). From the perspective of imbalance types, different regions exhibit differentiation characteristics: some new cities belong to the supply shortage type with demand growth and supply lag; Some old communities have an oversupply of idle facilities and wasted resources, and structural imbalance has become a common problem.

Meanwhile, the heterogeneity of older adult needs has gradually garnered attention. The current research on supply–demand matching of older adult care services has shifted from homogeneous measurement of the total older adult population to hierarchical identification of demand capacity, facility types, and travel modes. Sun et al. (3) used the proportion of urban older adult population to the total older adult population in the province as the demand side indicator, and the proportion of the number of older adult care facilities to the total number of facilities in the province as the supply side indicator. They constructed a consistency coefficient through the ratio of the two and divided the city into resource advanced, supply–demand matching, and resource lagging types, thereby identifying the relative deviation between the allocation of older adult care facilities and the distribution of older adult population. This method can reveal the resource allocation hierarchy in different regions, but its demand side still uses the total older adult population as the unified measurement object, and has not further distinguished the service demand differences of different age, health status, and mobility groups.

In terms of supply side stratification, Gu et al. (17) classified older adult care institutions, home-based older adult care service centers, and older adult canteens into different facility types based on facility levels, service functions, and service capabilities, and assigned comprehensive weights of 0.4, 0.3, and 0.3, respectively; In terms of travel modes, according to the survey results, the proportion of older adult people walking, public transportation, and driving is set at 63.2, 20.5, and 16.3%, with corresponding maximum search times of 15 min, 30 min, and 30 min, respectively. The attractiveness of facility levels, transportation modes, and distance attenuation are then included in the multi-mode two-step mobile search model. This method incorporates the service level of older adult care facilities and the differentiated travel modes of the older adult into a unified framework, breaking through the assumption of a unified facility type and a single travel radius. However, its population demand is still mainly characterized based on the spatial distribution of the older adult population, and the characterization of age structure and physical ability differences is still limited.

The more detailed stratification on the demand side is mainly reflected in the differences in the mobility of the older adult. Based on survey data and health tracking data, Ning classified older adult people into three categories: those with normal mobility, those with mild mobility restrictions, and those with severe mobility restrictions. The population proportions were 80, 16, and 4%, respectively, with corresponding walking speeds of 1.10 meters per second, 0.67 meters per second, and 0.44 meters per second. Under a unified 15 min walking time constraint, the maximum walking distances for the three groups are 1,000 meters, 600 meters, and 400 meters, respectively. Based on this, differentiated service ranges are constructed, and facility accessibility is calculated using a kernel density two-step mobile search method. Then, Gini coefficient, Lorenz curve, and location entropy are used to evaluate the fairness of resource allocation (21). This method indicates that the key to demand stratification is not simply to increase the population weight of older adult people with limited mobility, but to redefine the scope of facility services based on their actual walking speed and maximum travel distance; Adopting a unified service radius may overestimate the actual access to older adult care resources for individuals with mild and severe mobility restrictions. Previous studies have gradually progressed from regional total matching to facility level, transportation mode, and mobility stratification, providing a clear methodological path for identifying heterogeneity in older adult care service demand.

In summary, the current supply–demand matching calculation generally adopts a homogeneous assignment of the total older adult population in the entire region, without setting age specific demand weights based on differences in walking tolerance among different groups, which can easily lead to evaluation bias. There is a significant difference in acceptable walking radius between young healthy older adult and older adult or disabled groups, and a uniform threshold may underestimate the pressure of older adult care in old communities (3, 17). The existing evaluation system lacks sub indicators such as the older adult disability rate and the proportion of people with limited mobility. Introducing a hierarchical weighting approach can refine demand estimation and improve the accuracy of supply–demand adaptation assessment (21). There are significant differences in the demand for older adult care services among seniors of varying ages, health conditions, and income levels. The existing service provision system exhibits a tendency toward homogenization, further exacerbating the mismatch between supply and demand (22, 23). The current supply and demand measurement forms two mainstream paradigms: total homogenization and age weighted. Both have their own advantages and disadvantages in terms of data threshold and demand characterization accuracy. Existing cross-sectional studies only use one method alone, lacking horizontal comparison between the two schemes in the same research area. It is difficult to quantify the supply and demand evaluation bias caused by the stratified demand of the older adults, and it is also difficult to construct a comprehensive evaluation framework that takes into account population differences and multidimensional spatial characteristics.

Spatial equity research extends this discussion further, using Gini coefficients and spatial autocorrelation to evaluate equality of older adults care distribution (12, 24). Inequality persists nationwide, characterized by multi-core concentration and persistent peripheral deficiency. Gu Mingxia et al. (2025) (17) demonstrated significant urban–rural disparities, while Lu Guangyu et al. (2026) (10) and Hu Haohao et al. (2025) (20) proposed optimization strategies through resource redistribution and cross-regional sharing. The Gini coefficient measures the overall equity of resource distribution across a region, while the spatial autocorrelation model identifies localized patterns of supply–demand imbalance. These two approaches complement each other in evaluation dimensions; however, most existing studies rely solely on individual indicators, lacking integrated calculations or cross comparisons of multidimensional fairness outcomes. Existing literature has depicted the reality of uneven distribution of older adults care resources from multiple dimensions, but an integrated analysis framework that can coordinate facility spatial structure, accessibility differentiation, and supply–demand adaptation level has not yet been formed. Various empirical conclusions are fragmented, making it difficult to provide accurate planning policy references for mega cities like Guangzhou with complex spatial structures and uneven development.

### 2.4 Research gaps and analytical implications

Overall, spatial research on older adults care service facilities in China has achieved considerable methodological richness and empirical depth, covering topics such as spatial layout identification, network accessibility evaluation, and supply–demand coordination analysis These studies demonstrate that the allocation of older adults care resources across different cities follows common spatial patterns and exhibits significant regional disparities. The existing research is divided into three independent branches: layout, accessibility, and supply and demand. Each branch uses dedicated measurement tools and evaluation logic, and lacks horizontal comparison and element linkage analysis between branches; The existing achievements are mostly single dimensional cross-sectional evaluations, which generally ignore the demand stratification differences caused by age and walking ability of the older adults, and cannot integrate multi-source data to form an integrated research and judgment system, making it difficult to fully explain the compound driving mechanism of urban older adults care resource imbalance.

However, existing research still has shortcomings such as insufficient theoretical integration and limited depth of comprehensive evaluation. Various types of older adults care facilities are often separated and analyzed independently, and the collaborative service logic between institutions, communities, and dietary assistance facilities is difficult to fully present. Many studies have separately explored the correlation between population aging and facility distribution, but lack a unified analysis framework that can integrate multi-source data on population, facilities, and accessibility, making it difficult to fully interpret the internal logic of supply and demand adaptation of older adults care resources. Previous studies have mostly relied on single dimensional models to describe cross-sectional spatial features, with various analysis tools used independently and lacking a multi method linkage analysis approach, making it difficult to fully present the linkage relationships between facilities, population, and transportation networks.

The refined evaluation leveraging POI big data and GIS models still has room for improvement. There are few studies that have conducted comprehensive multi-indicator assessments of supply–demand alignment across entire megacities, and local service gaps within cities remain difficult to identify precisely. Relevant research provides limited support for urban planning in cities like Guangzhou, which exhibit significant urban–rural development disparities and complex spatial structures. Single-dimensional analytical approaches sever the intrinsic connections between facility distribution, pedestrian accessibility, supply–demand matching, and spatial equity, hindering the development of integrated optimization strategies covering the entire area with layered categorization. While existing empirical findings from various cities offer foundational references for this study, their differing research perspectives and calculation methods make it challenging to directly support holistic urban older adult care space planning.

Based on this, this article selects 11 administrative districts in Guangzhou as the research scope, integrating GIS spatial analysis tools, road network pedestrian accessibility measurement, and supply–demand adaptability evaluation models into a complete multidimensional analysis framework. Research on synchronously sorting out the spatial distribution pattern of facilities, regional accessibility differences, and the internal logic of supply and demand coordination of older adult care resources, systematically analyzing the spatial differentiation of public older adult care services and the fairness of resource allocation, in order to provide empirical basis and planning decision support for optimizing the allocation of older adult care service resources and improving the overall efficiency and balance level of supply in large cities in China.

## 3 Research design and methods

### 3.1 Overview of the research area

Located in the central and southern part of Guangdong Province and at the core of the Pearl River Delta, Guangzhou is a national central city and a key driving force of the Guangdong–Hong Kong–Macao Greater Bay Area. The city covers an area of approximately 7,434 km^2^ and administers 11 districts: Yuexiu, Liwan, Haizhu, Tianhe, Baiyun, Huangpu, Panyu, Huadu, Nansha, Conghua, and Zengcheng. As of the end of 2024, Guangzhou’s permanent population reached about 18.97 million, ranking among China’s largest megacities. With the continued aging of the population, the number of older adult residents has been rising rapidly, and the demand for older adult care services is growing accordingly. Owing to historical development patterns, population distribution, and differing levels of economic growth, there are marked disparities in the degree of aging, the concentration of older adult residents, and the allocation of older adult care resources across districts.

As one of China’s pilot cities for reforms in home and community-based older adult care, Guangzhou has actively promoted the development of its older adults care service system, gradually forming a multilevel network that includes institutional care, community care, meal assistance, medical-care integration, and comprehensive service provision. Nevertheless, under accelerating population aging and increasing demands, questions remain over whether resources are optimally matched to spatial demand and whether older adults residents enjoy equitable access. Therefore, taking Guangzhou as the study area and analyzing the spatial distribution, supply–demand coordination, and accessibility of public older adults care facilities provides an important empirical foundation for improving resource allocation and promoting balanced service development.

### 3.2 Data sources

To measure the accessibility and supply–demand adaptability of public older adults care service facilities in Guangzhou, this article refers to the existing research paradigm of urban older adults care service supply and demand to construct an evaluation index system (25, 26). In terms of indicator selection, the demand side mainly reflects the regional demand for older adult care and the burden of older adult care by comprehensively considering the size of the older adult population, the degree of aging, and the older adult dependency ratio; On the supply side, Feng et al. (10) drew on institutional older adults care facilities, community older adults care facilities, and meal assistance service facilities as the core, while introducing the density of medical services and public service facilities as supporting variables to construct a multidimensional supply index system that comprehensively reflects the supply capacity of public older adults care services in Guangzhou. Similar urban‑scale empirical research also provides practical references for constructing evaluation indicators for community and disability‑targeted institutional care facilities (28, 29). Considering that this study focuses on the fairness of spatial allocation of public older adults care service resources, the supply side indicators are mainly measured from the dimension of facility spatial supply. The number of facilities and POI density can better reflect the spatial distribution characteristics and service coverage level of public older adult care service facilities, and have been widely used in the research of public service facility layout and spatial accessibility. The density of various facilities on the supply side is based on the basic analysis unit of each administrative district in Guangzhou, with the land area (km^2^) of each administrative district as the standardized benchmark. It is calculated by dividing the number of POIs of corresponding types of public older adults care service facilities in each administrative district by the land area of the administrative district. This is used to characterize the spatial distribution density of older adults care service facilities in different regions, as shown in Table 1.

**Table 1:** Index system for the adaptability of supply and demand of public older adults care service facilities in Guangzhou.

System level	Indicator level	Content	Unit
Demand for older adults care services (demand side)	Older adults population density	Population aged 60 and above per administrative region/Land area of each administrative region	Person/km^2^
	Aging degree	Proportion of population aged 60 and above in the total population	%
	Old-age dependency ratio	Older adults population/working age population	%
Scale of public older adults care service facilities (supply side)	Density of institutional older adult care facilities	Number of POIs (Points of Interest) for older adults care facilities per administrative region/Land area of each administrative regions	One per square kilometer
	Density of community older adults care facilities	Number of POIs (Points of Interest) for community-based older adults care facilities per administrative region/Land area of each administrative region	One per square kilometer
	Density of meal service facilities	Number of POIs (Points of Interest) for meal assistance services per administrative region/Land area of each administrative region	One per square kilometer
	Density of medical service facilities	Number of medical service facilities (POIs) per administrative region/Land area of the administrative region	One per square kilometer
	Density of public service facilities	Number of public service facilities (POIs) per administrative region/Land area of the administrative region	One per square kilometer
	Number of nursing beds per thousand older people	Number of nursing beds/older population × 1,000	One bed per thousand people

This study collected relevant data from Guangzhou City and 11 districts in 2024 based on an indicator system. The data on older adults care demand mainly comes from the “Guangzhou Statistical Yearbook” and the statistical bulletins of each district, which are the standard data sources for regional older adults care demand calculation. The data of public older adults care service facilities is obtained based on Baidu Maps POI data, which has become the mainstream database for spatial research of urban public service facilities (5, 27). Furthermore, this study systematically categorizes and organizes POI data related to older adults care facilities. Using the list of such facilities published by Guangzhou’s Comprehensive Older adult Care Service Platform as a reference, the data underwent classification verification, removal of duplicate entries, and spatial position correction to ensure accuracy and completeness; details are presented in Table 2.

**Table 2:** POI data explanation of public older adults care service facility types in Guangzhou City.

Service Facility Type	POI Type	Quantity
Institutional older adults care facilities	Nursing homes, nursing homes, nursing homes, senior apartments, sanatoriums, etc. are institutions that provide centralized living and care services for the older adults	255
Community older adults care facilities	Community older adults care service centers, day care centers, daycare centers, and older adults activity centers are facilities that provide day care, rehabilitation, entertainment, and other services for older people living at home	2,172
Meal service facilities	Facilities such as senior dining halls, community assisted meals, and senior canteens that provide catering support and meal preparation services for the older adults	1,230
Medical service facilities	Comprehensive hospitals, specialized hospitals, community health service centers, clinics, pharmacies, and other facilities that provide disease diagnosis, treatment, health management, and rehabilitation services for the older adults	9,609
Public service facilities	Facilities provided by government agencies, community service centers, social service institutions, etc. to provide social security and public service support for the older adults	4,543

### 3.3 Research methods

#### 3.3.1 GIS spatial analysis method

Spatial statistical analyses were conducted in ArcGIS using Average Nearest Neighbor (ANN), Kernel Density Estimation (KDE), and Standard Deviation Ellipse (SDE) methods to describe clustering, density, and directional trends of public older adult care facilities.

##### 3.3.1.1 Average nearest neighbor (ANN)

Average Nearest Neighbor (ANN) identifies the spatial clustering characteristics of public older adults care facilities in Guangzhou by comparing the actual average nearest neighbor distance of older adults care facilities with the theoretical average nearest neighbor distance under random distribution conditions. The calculation Equation 1 is as follows:

$$\mathrm{ANN}=\frac{{\bar{r}}_{i}}{{\bar{r}}_{E}}={\bar{r}}_{i}\times 2\sqrt{D}={\bar{r}}_{i}\times 2\sqrt{\frac{n}{A}}$$ (1)

In the formula, ANN represents the nearest neighbor index; ${\bar{r}}_{i}$ represents the actual average nearest neighbor distance; ${\bar{r}}_{E}$ represents the theoretical average nearest neighbor distance under random distribution conditions; *D* represents the density of older adults care service facilities; *n* is the number of older adults care service facilities; *A* is the area of the research region. When ANN < 1, it indicates that the older adults care service facilities are distributed in agglomeration; when ANN = 1, they are distributed randomly; when ANN > 1, they are distributed uniformly.

Meanwhile, significance tests were conducted on spatial distribution characteristics using Z-test and *p*-value test. When *p* < 0.05, it indicates that the spatial distribution characteristics have passed the significance test; When the Z value is greater than 0, it indicates that public older adult care service facilities are spatially clustered; When the Z value is less than 0, it indicates a spatially discrete distribution; When the Z value approaches 0, it indicates that the spatial distribution tends to be random. The larger the absolute value of Z, the higher the degree to which the spatial distribution deviates from the random state, and the more significant its spatial clustering or discretization characteristics.

##### 3.3.1.2 Kernel density estimation (KDE)

KDE identifies hotspots of facility concentration by calculating density within a defined search radius. Higher kernel density values indicate stronger facility clusters per unit area, whereas lower values signal spatial dispersion. The calculation Equation 2 is as follows:

$$\mathrm{KDE}=\sum_{i=1}^{n}[P_{i}\times \frac{1}{h^{2}}\times K(\frac{d_{i}}{h})]$$ (2)

In the formula, KDE represents the kernel density value; *P_i_* represents the weight value of the i-th older adults care service facility point, and when the weight is not considered, the value is 1; h represents the search radius; di represents the distance from the i-th older adults care service facility point to the location where the core density is calculated. The higher the kernel density value, the higher the degree of aggregation of public older adults care service facilities in Guangzhou per unit space; The lower the nuclear density value, the more dispersed the facility distribution is.

##### 3.3.1.3 Standard deviation ellipse (SDE)

Standard Deviation Ellipse (SDE) reveals the spatial distribution direction and expansion trend of public older adults care facilities in Guangzhou by measuring the average center, dispersion degree, and dominant direction of spatial elements. The calculation Equations 3–5 are as follows:

$$\bar{X}=\frac{\sum_{i=1}^{n}X_{i}}{n},\bar{Y}=\frac{\sum_{i=1}^{n}Y_{i}}{n}$$ (3)

In the formula, $\left(\bar{X},\bar{Y}\right)$ represents the average center coordinate of the distribution of older adult care service facilities; *X_i_* and *Y_i_* are the spatial coordinates of the i-th older adult care service facility point.

$$\sigma_{x}=\sqrt{\frac{\sum_{i=1}^{n}{\left(X_{i}-\bar{X}\right)}^{2}}{n}},\sigma_{y}=\sqrt{\frac{\sum_{i=1}^{n}{\left(Y_{i}-\bar{Y}\right)}^{2}}{n}}$$ (4)

In the formula, $\sigma_{x}$ represents the standard deviation along the long axis direction; $\sigma_{Y}$ represents the standard deviation along the short axis direction.

$$\tan\theta =\frac{(\sum x_{i}^{2}-\sum y_{i}^{2})+\sqrt{(\sum x_{i}^{2}-\sum y_{i}^{2})+4{\left(\sum x_{i},Y_{i}\right)}^{2}}}{2\sum x_{i}Y_{i}}$$ (5)

In the formula, θ is the rotation angle of the standard deviation ellipse, reflecting the dominant direction of spatial distribution of public older adult care service facilities in Guangzhou.

#### 3.3.2 Spatial accessibility evaluation

Accessibility reflects how easily older adults residents can reach care resources. Because these facilities mainly serve older adult, accessibility depends not only on facility quantity and spatial layout but also on the efficiency of transportation networks. In recent years, the two-step floating search method (2SFCA) and its extended models have been widely used in research on the accessibility of medical and older adults care services. This method can comprehensively consider facility supply capacity, demand scale, and distance attenuation effects. The focus of this study is to evaluate the actual walking accessibility level of the older adults population along the road network to reach older adults care service facilities, and further analyze the allocation of older adults care service resources by combining supply and demand adaptation models. Therefore, a network accessibility analysis method based on the shortest path of the road network is adopted to truly reflect the walking time and spatial coverage characteristics of the older adults population along the road network to reach the older adults care service facilities. To evaluate the spatial accessibility level of public older adult care service facilities in Guangzhou, this paper constructs a road network dataset based on ArcGIS Network Analyst module. The walking time of the older adults is taken as the impedance factor, and the shortest path algorithm is used to calculate the network travel time from residential areas to older adults care service facilities. The calculation Equation 6 is as follows:

$$T_{\mathrm{ij}}=\frac{L_{\mathrm{ij}}}{V}$$ (6)

In the formula, *T*_ij_ represents the walking time (in minutes) from residential area *i* to older adults care service facility *j*; *L*_ij_ represents the shortest path distance (m) from residential area i to older adults care service facility *j*; *V* represents walking speed (m/min) and the travel characteristics of the older adults are taken as 3.5 km/h (ie. 58.3 m/min). Establish 15-min, 30-min, and 60-min older adults care service zones to identify the spatial coverage characteristics of older adults care facilities and areas with service gaps.

#### 3.3.3 Adaptability model

This paper develops an evaluation model for supply–demand compatibility based on two dimensions—coordination and matching—to measure the alignment between public older adults care service demand and supply in Guangzhou.

1. Coordination measurement.

The coordination index measures the degree of coordinated development between Guangzhou’s public older adult care demand system and supply system, calculated using a coupling coordination model as shown in Equations 7–9:

$$T=a_{1}S_{1}+a_{2}S_{2}$$ (7)

In the formula, *T* represents the comprehensive development level of the demand and supply system for older adults care services, which is calculated using the entropy method in this article; *S*_1_ and *S*_2_, respectively, represent the demand index and supply index for older adults care services; *a*_1_ and *a*_2_ are weight coefficients. Considering that the demand and supply systems have equal importance in the older adults care service system, a1 = a2 = 0.5 is taken.

$$c=\sqrt[{n}]{\frac{\prod_{i=1}^{n}S_{i}}{{\left(\frac{1}{n}\sum_{i=1}^{n}S_{i}\right)}^{n}}}$$ (8)

In the formula, C represents the coupling degree between the demand system and the supply system of older adults care services, indicating the degree of correlation between the demand and supply of older adults care services. The range of values is [0, 1], and the closer the value is to 1, the stronger the correlation.

$$D=\sqrt{C\times T}$$ (9)

In the formula, *D* represents the coordinated development level between the demand and supply system of older adults care services, indicating the degree of coordination and adaptation between the demand and supply of older adults care services. The range of values is [0, 1], and the closer the value is to 1, the higher the level of coordinated development.

##### 3.3.3.1 Matching measurement

Matching reflects the degree of consistency between the demand and supply scale of older adults care services in Guangzhou, measured by the difference in ranking between the two systems. The calculation formula is as follows:

$$M_{j}=1-\frac{|W_{j}|}{n-1}$$ (10)

In the formula, *M_j_* represents the supply–demand matching of the j-th administrative region; w*_j_* represents the difference in ranking between the demand index and supply index for older adult care services; n is the number of research units, and this study takes 11 administrative districts in Guangzhou city; The value range of *M_j_* is [0, 1], and the closer the value is to 1, the higher the degree of supply–demand matching.

##### 3.3.3.2 Adaptability measurement

$$A_{j}=aD_{j}+bM_{j}$$ (11)

In the calculation Equation 11, *A_j_* represents the level of supply–demand adaptability of older adults care services in the j-th administrative region; a and b are the weights representing coordination degree and matching Given that coordination index reflects the level of coordinated development between older adult care demand and supply systems, while matching index indicates the equilibrium relationship between supply and demand, the former holds greater significance in comprehensive evaluation. Therefore, referencing relevant literature (23, 24), weights of 0.6 for coordination index and 0.4 for matching index were assigned. To validate the rationality of these weight settings, sensitivity analyses were conducted using different weight configurations, which demonstrated consistent conclusions across all scenarios, indicating robustness of the proposed weighting framework. The adaptability of *A_j_* is divided into five levels, namely high adaptability, relative adaptability, relatively compatible, unsuitability, and extremely unsuitability, to reveal the spatial differentiation characteristics of older adult care service resource allocation, as shown in Table 3.

**Table 3:** Classification of adaptability levels.

Adaptability range	Adaptability level
0.0000≦A≦0.3999	Very low suitability
0.4000≦A≦0.5999	Low suitability
0.6000≦A≦0.6999	Moderate suitability
0.7000≦A≦0.7999	High suitability
0.8000≦A≦1.0000	Very high suitability

## 4 Analysis of empirical results

### 4.1 Spatial distribution characteristics of supply and demand for older adult Care Services in Guangzhou

#### 4.1.1 Spatial distribution characteristics of older adult care demand in Guangzhou

To analyze the spatial differences in demand for older adult care services in Guangzhou, this article selects three indicators: older adult population density, aging degree, and older adult dependency ratio. The analysis is conducted from three dimensions: demand scale, population aging level, and older adult care burden pressure. The results are shown in Table 4 and Figure 1.

**Table 4:** The demand for older adults care services in various districts of Guangzhou.

Regional	Older adults population density (person/km^2^)	Aging degree (%)	Older adults dependency ratio (%)
Liwan district	4,353	31.61	61.81
Yuexiu district	10,388	30.14	56.18
Haizhu district	3,632	29.42	55.14
Tianhe district	1847	16.08	26.42
Baiyun district	279	17.6	30.72
Huangpu district	200	13.13	22.69
Panyu district	343	14.7	25.07
Huadu district	151	15.81	27.54
Nansha district	121	16.28	28.09
Conghua district	53	15.68	26.34
Zengcheng district	105	14.78	25.63

**Figure 1:**
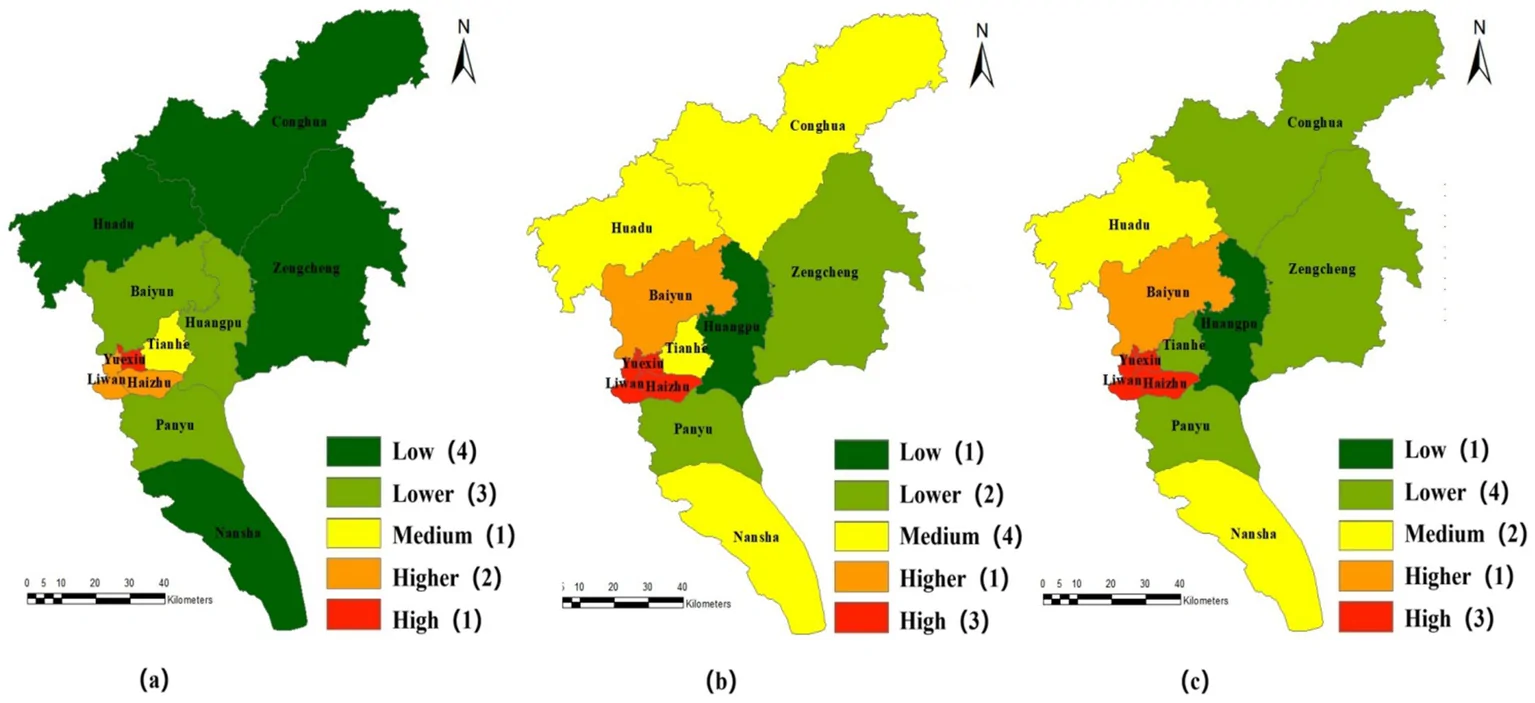
Spatial distribution of older adults care service demand in Guangzhou City **(a)** Older adults population density. **(b)** Aging degree. **(c)** Older adults dependency ratio.

The older adult population density in Guangzhou shows distinct spatial clustering. Yuexiu District records the highest older adult population density at 10,388 people/km^2^, followed by Liwan District (4,353 people/km^2^) and Haizhu District (3,632 people/km^2^), jointly forming a highly concentrated zone of older adult residents. Tianhe District falls at a moderate level, while Baiyun, Huangpu, Panyu, Huadu, Conghua, and Zengcheng Districts all show relatively low densities. Overall, a spatial pattern emerges with Yuexiu District as the core, radiating outward to Liwan and Haizhu Districts and gradually declining toward the periphery. As traditional central urban areas, Yuexiu, Liwan, and Haizhu Districts feature early urban development, high population density, and mature residential communities that have retained older adult residents over time. Peripheral districts, by contrast, cover larger land areas and maintain lower population densities, resulting in a more dispersed older adult population.

In terms of the level of aging, Guangzhou exhibits a clear spatial differentiation characterized by a high-center and low-periphery pattern. Aging rates in Liwan, Yuexiu, and Haizhu Districts all exceed 29%, with Liwan District reaching 31.61%, the highest in the city. In contrast, aging levels in Huangpu, Panyu, and Zengcheng Districts are below 15%. Spatially, high-value areas are concentrated in the traditional central urban districts, forming a continuous core of aging, while outer districts display low to medium levels. This pattern reflects the cumulative historical effect of population aging. Limited housing renewal and low residential mobility have led to a concentration of older adults residents in central areas, while younger populations have moved to emerging suburban districts such as Huangpu and Panyu, drawn by industrial development and new housing supply.

The spatial distribution of the older adults dependency ratio mirrors that of the aging level. Liwan, Yuexiu, and Haizhu Districts have dependency ratios of 0.6181, 0.5618, and 0.5514, respectively—far higher than in other districts—indicating a heavier older adults-care burden on the working-age population. In Huangpu, Panyu, and Zengcheng, the ratio remains relatively low, corresponding to a younger population structure. The overall gradient decreases from the central urban region toward the periphery, suggesting that the central districts not only experience stronger demand pressure for older adults care services but also face greater social and economic responsibility for older adults support.

Synthesizing the three demand indicators reveals a pronounced spatial imbalance in Guangzhou’s older adults care service needs. Old urban areas demonstrate high demand aggregation, whereas peripheral districts exhibit dispersed demand. Yuexiu, Liwan, and Haizhu Districts represent the city’s core zones of concentrated older adults-care demand across all indices. Tianhe District, although hosting a large older adults population, maintains a relatively low aging rate and a strong capacity to attract younger residents. Peripheral areas such as Huadu, Conghua, Zengcheng, and Nansha Districts have lower overall demand levels, yet their broad geographic extent and scattered communities pose challenges of long service radii and limited coverage for facility planning.

#### 4.1.2 Spatial distribution characteristics of older adults care service supply in Guangzhou

To further reveal the spatial organization patterns of public older adults care facilities, this study applies the nearest-neighbor index, kernel-density analysis, and standard-deviation ellipse methods. The comprehensive results are presented in Table 5 and Figure 2.

**Table 5:** Nearest neighbor index (ANN) of various types of public older adults care service facilities in Guangzhou City.

Facility	Nearest neighbor ratio	Average observation distance(m)	Predicted average distance(m)	*Z*	*p*
Institutional older adults care facilities	0.567324	1788.9396	3153.2933	−13.217934	0.000000
Community older adults care facilities	0.273560	320.4215	1171.3036	−64.768033	0.000000
Meal service facilities	0.459607	727.7814	1583.4880	−35.647731	0.000000
Medical service facilities	0.247843	146.5414	591.2675	−141.051785	0.000000
Public service facilities	0.135841	106.9906	787.6173	−111.428425	0.000000

**Figure 2:**
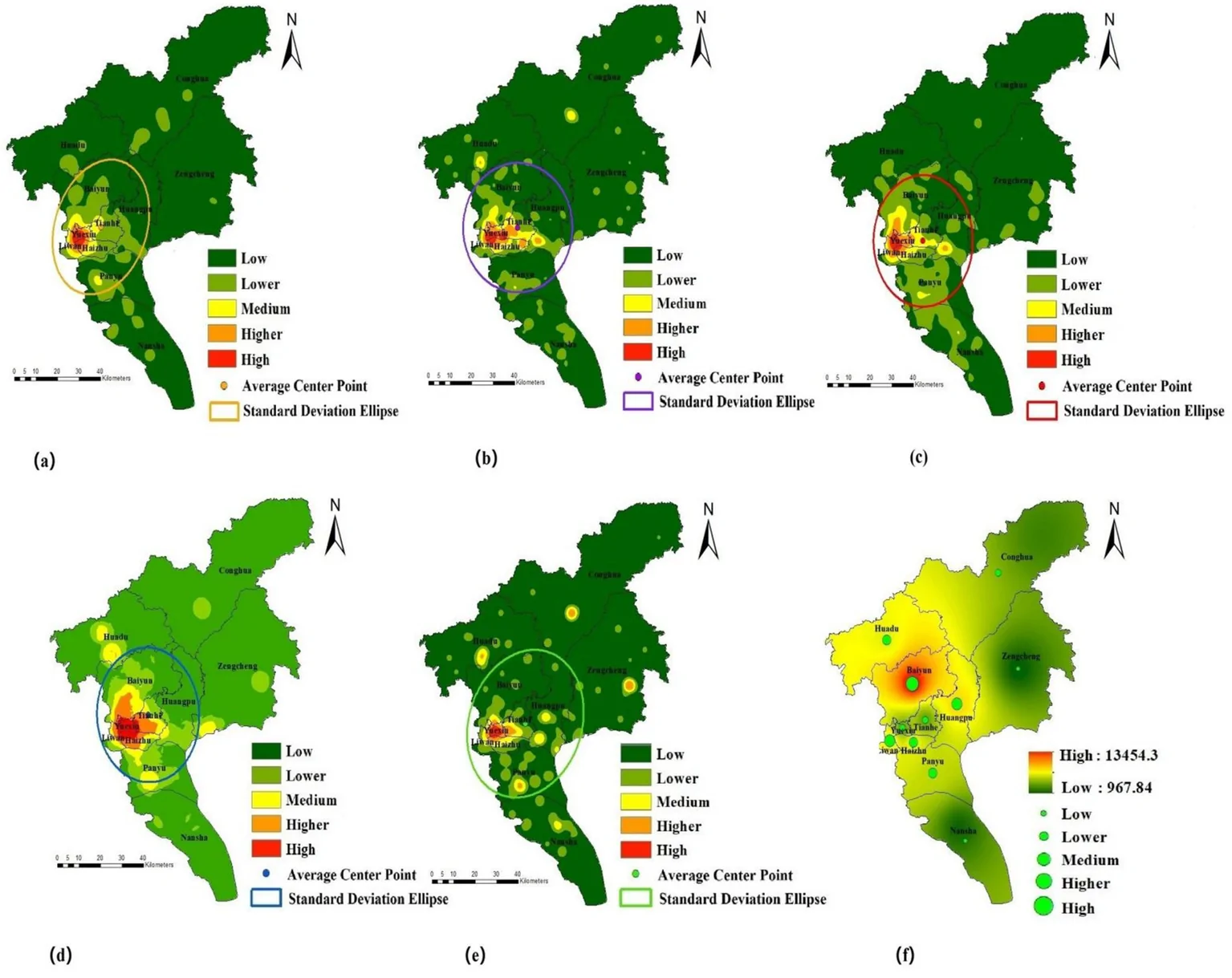
Spatial distribution map of various types of public older adult care service facilities in Guangzhou. **(a)** Kernel Density of Institutional Older adult Care Facilities. **(b)** Kernel Density of Community Older adult Care Facilities. **(c)** Kernel density of meal service facilities. **(d)** Kernel density of medical service facilities. **(e)** Kernel density of public service facilities. **(f)** Kernel density of older adult care beds.

As shown in Table 5, the ANN values for all types of older adults care service facilities are below 1, and the associated *p*-values are all less than 0.001, indicating statistically significant clustering across facility categories. Public service facilities show the smallest nearest-neighbor value (0.1358), followed by community older adults care facilities (0.2736) and medical service facilities (0.2478), implying that these three types exhibit the strongest spatial clustering. Institutional older adults care facilities have a higher value (0.5673), suggesting a comparatively dispersed distribution. Moreover, the observed average distances of all facility types are notably smaller than their expected values under theoretical random conditions, further confirming the pronounced clustering pattern of older adults care service facilities in Guangzhou.

According to the kernel density maps in Figures 2a–e, all facility types display clustering centered around the core urban area, though clustering intensity and diffusion range vary. Institutional older adults care facilities mainly concentrate along the boundaries of Liwan, Yuexiu, Haizhu, and Tianhe Districts, extending southward into Baiyun and northward into Panyu. Community older adults care and meal-assistance facilities exhibit stronger high-density clustering, with hotspot areas primarily located in Yuexiu, Liwan, and Haizhu Districts and extending westward into Tianhe District. This reflects a strong spatial coupling between facility locations and densely populated neighborhoods. Medical service and public service facilities show spatial patterns similar to community older adults care centers, with high-density zones concentrated in the central urban area and forming a contiguous core cluster. This distribution demonstrates coordinated resource allocation in developing integrated medical and older adults care systems and comprehensive service networks.

The standard-deviation ellipse results in Figures 2a–e indicate that the mean centers of all facility types are situated within the central urban area. The ellipses extend predominantly along a northeast–southwest axis, aligning closely with both Guangzhou’s population distribution and its main development corridor. The ellipse of institutional older adults care facilities covers the largest area—spanning parts of Baiyun, Panyu, and Huangpu Districts—signifying a wide service radius and broad spatial coverage. Other older adults-care facilities show tighter, more centralized ellipses around the core urban zones, suggesting a greater focus on proximity and community-embedded service provision.

From Figure 2f, it can be seen that the spatial distribution of older adults care bed resources exhibits obvious spatial clustering characteristics, but there are certain differences between their clustering centers and the spatial clustering of various older adults care service facilities. The high-value areas are mainly distributed in the border area between the southern part of Baiyun District and the western part of Huangpu District, and extend to the southeastern part of Huadu District, forming a relatively continuous area of bed resource aggregation; The low value areas are mainly distributed in peripheral areas such as Conghua District, Zengcheng District, and Nansha District. Overall, the resources of older adults care beds are not completely dependent on the central urban area, but rather form new spatial clusters on the outskirts of the city.

### 4.2 Spatial accessibility characteristics of public older adults care service facilities in Guangzhou

To further evaluate the rationality of Guangzhou’s spatial configuration of public older adults care facilities, network-analysis techniques were applied based on the city’s road-network and facility-location data. Walking time to the nearest facility was calculated, and accessibility was categorized into four levels: 0–15 min, 15–30 min, 30–60 min, and above 60 min. The results are shown in Figure 3.

**Figure 3:**
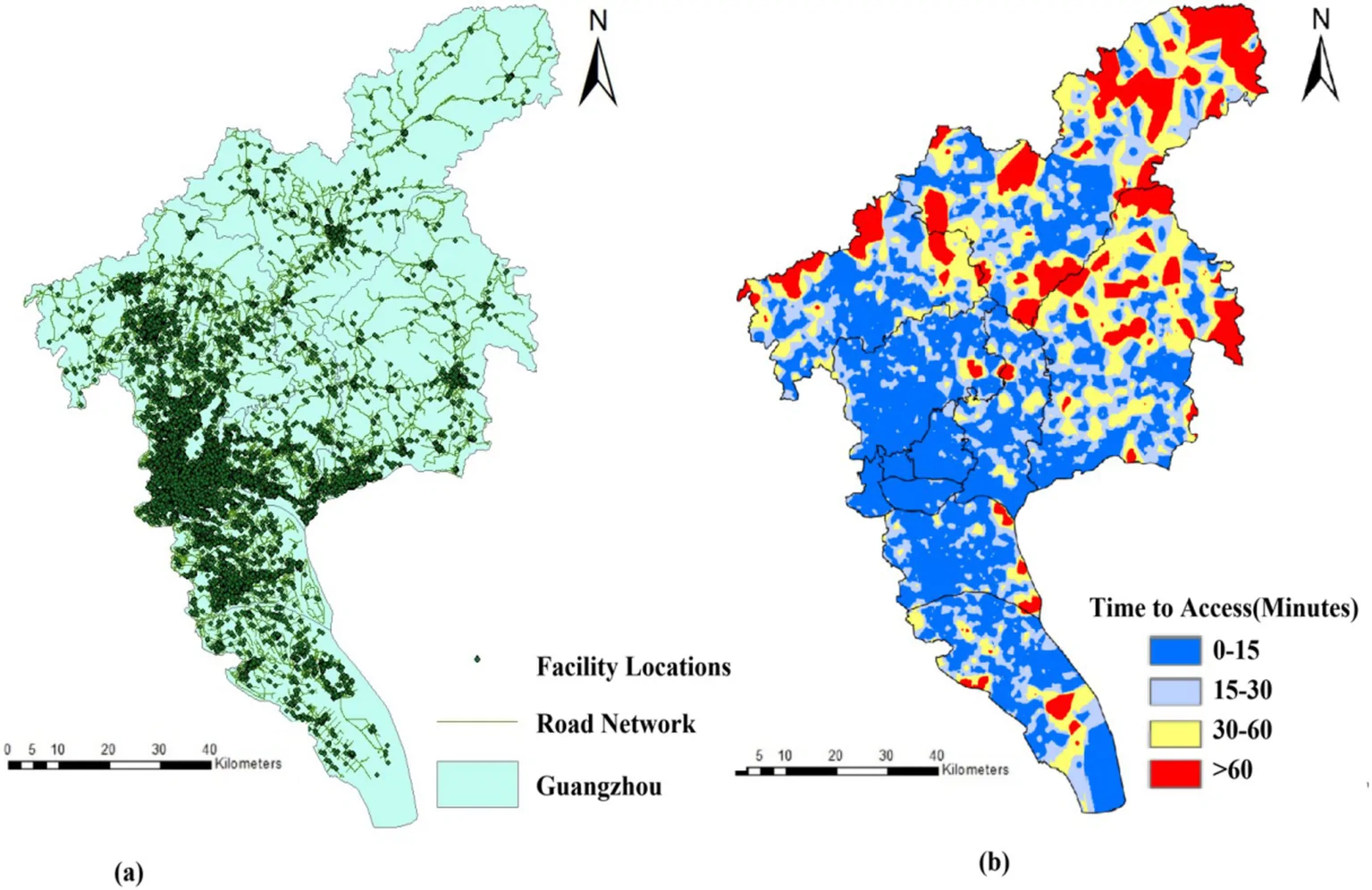
Spatial accessibility map of public older adult care service facilities in Guangzhou City. **(a)** Facility locations and road network. **(b)** Facility Accessibility.

From the distribution of facilities and the road network in Figure 3a, it is evident that the spatial layout of public older adult care facilities corresponds closely with the city’s transport infrastructure. Most facilities are located along major transport corridors, reflecting a strong road-network orientation. This indicates that the structure and density of the transportation network play a crucial role in shaping both spatial coverage and service accessibility of older adult care resources.

According to the spatial accessibility patterns in Figure 3b, the overall accessibility of public older adult care facilities in Guangzhou is high: most of the urbanized area lies within a 15-min walking range, forming a concentric pattern radiating from the central city outward. Yuexiu, Liwan, Haizhu, Tianhe, and northern Panyu Districts largely fall within the 0–15-min zone, meaning residents in these districts can access older adults care services conveniently and efficiently. Their high accessibility arises from dense populations, well-developed transport systems, and compact facility layouts.

Areas reachable within 15–30 min are distributed in a ring surrounding the core, encompassing most of Baiyun, Huangpu, Huadu, and Zengcheng Districts. Facilities in these zones can generally meet basic service needs, but their coverage and efficiency are weaker than those of the inner-city areas, and residents face higher time costs. The 30–60 min zones occur mainly on the urban fringes and within several urban–rural transition belts, where facility density and road accessibility are both relatively low.

Regions requiring more than 60 min of travel are concentrated in northern Conghua, northeastern Zengcheng, and several mountainous or hilly edge areas. These zones feature rugged terrain, scattered settlements, and weaker transportation links. Limited facility distribution further increases travel times, creating pockets of service deficiency. However, because population density in these peripheral and mountainous areas is low, their impact on the citywide accessibility pattern remains limited. Overall, Guangzhou demonstrates a graded accessibility structure that weakens from the urban center toward the periphery, shaped primarily by the combined effects of population distribution, topography, and transport conditions.

### 4.3 Spatial variability characteristics of supply–demand compatibility in Guangzhou’s public older adults care services

#### 4.3.1 Analysis of the Matchability between supply and demand of public older adults Care Services in Guangzhou

To examine the degree of coordination between public older adults care service supply and population demand, a comprehensive supply–demand adaptability evaluation system was established using the respective supply and demand indices. The adaptability model was combined with Local Indicators of Spatial Association (LISA) cluster analysis to measure both the overall adaptation level and its spatial differentiation across districts. The analytical results are summarized in Table 6 and Figure 4.

**Table 6:** Adaptability and level of supply and demand of public older adults care services in Guangzhou City.

Regional	Older adults care service demand value	Older adults care service supply value	Coupling degree C value	Coordination index T value	Coordination degree D value	Matching	Adaptability	Grade
Liwan district	0.7201	0.5780	0.9940	0.6491	0.8032	1	0.8819	Very high suitability
Yuexiu district	0.9407	0.9218	0.9999	0.9313	0.9650	1	0.9790	Very high suitability
Haizhu district	0.6104	0.3499	0.9625	0.4802	0.6798	1	0.8079	Very high suitability
Tianhe district	0.1484	0.3424	0.9186	0.2454	0.4748	1	0.6849	Moderate suitability
Baiyun district	0.1262	0.1267	1	0.1265	0.3556	1	0.6134	Moderate suitability
Huangpu district	0.0069	0.1258	0.4440	0.0664	0.1716	0.5	0.3030	Very low suitability
Panyu district	0.0510	0.0750	0.9817	0.0630	0.2487	0.8	0.4692	Low suitability
Huadu district	0.0742	0.0548	0.9886	0.0645	0.2525	0.9	0.5115	Low suitability
Nansha district	0.0829	0.0279	0.8681	0.0554	0.2193	0.7	0.4116	Low suitability
Conghua district	0.0594	0.0195	0.8627	0.0395	0.1845	0.8	0.4307	Low suitability
Zengcheng district	0.0450	0.0068	0.6754	0.0259	0.1323	0.9	0.4394	Low suitability

**Figure 4:**
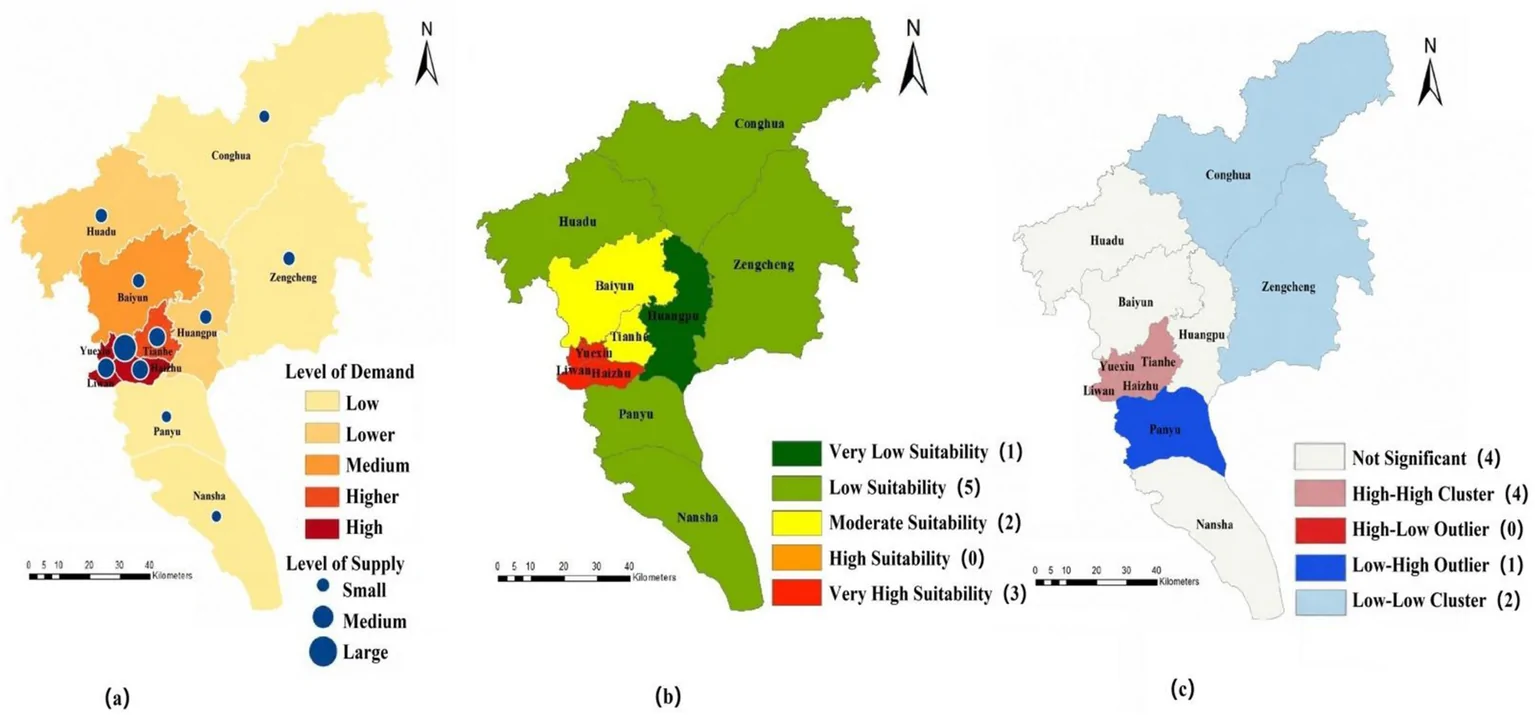
Spatial differentiation characteristics of supply and demand adaptability of public older adults care services in Guangzhou. **(a)** Demand-supply distribution **(b)** Suitability levels. **(c)** LISA cluster type.

As shown in Table 6, substantial regional variation exists in supply–demand relationships. Yuexiu, Tianhe, and Haizhu Districts show the highest adaptability indices—0.9790, 0.8819, and 0.8079—indicating a high degree of fit between supply and demand. Tianhe (0.6849) and Baiyun (0.6134) Districts demonstrate moderate adaptability, while several other districts display varying degrees of imbalance. Huangpu District, with an index of 0.3030, shows the lowest level of fit and a pronounced mismatch between facility provision and service demand. Overall, the spatial trend exhibits a gradual decline in adaptability from the central urban area to outer districts.

The spatial pattern presented in Figures 4a,b indicates that areas of high adaptability are mainly located in traditional central districts—Yuexiu, Liwan, and Haizhu—forming a continuous core of strong coordination between supply and demand. These areas combine high population demand with a rich concentration of community care centers, medical facilities, and public service resources, maintaining a dynamic balance. Tianhe and Baiyun Districts lie at the edge of this core and exhibit a transitional, gradient pattern of diffusion toward the periphery. In contrast, peripheral districts—Zengcheng, Conghua, Nansha, Panyu, and Huadu—generally fall within low-adaptability or maladaptive zones, revealing gaps between aging-population needs and facility allocation.

Local spatial autocorrelation analysis provides additional insight into clustering characteristics of adaptation levels. As shown in Figure 4c, Yuexiu, Tianhe and Haizhu Districts form significant high-high clusters, meaning not only do these areas exhibit high adaptability, but their neighboring districts also share consistently high values—demonstrating a strong positive spatial spillover effect. Zengcheng and Conghua Districts display low-low clusters, indicating low adaptability both locally and in adjacent areas and forming a relatively stable low-value lock-in pattern. Panyu District shows a low-high (heterogeneous) clustering type, suggesting that while its internal adaptability is low, neighboring areas exhibit higher adaptation levels, implying ample potential for targeted facility optimization and cross-regional resource coordination.

#### 4.3.2 Weight sensitivity analysis

To examine the impact of weight settings in the supply–demand matching model on research outcomes, this study employed three alternative weight configurations (0.5, 0.5; 0.7, 0.3; and 0.4, 0.6) and compared them with the baseline configuration (0.6, 0.4). As shown in Table 7, under all weight schemes, the calculated supply–demand matching indices for each administrative region maintained high consistency with the baseline values, exhibiting Pearson correlation coefficients of 0.9968, 0.9976, and 0.9854, respectively, (*p* < 0.001). Furthermore, the supply–demand matching rankings remained unchanged across all regions; only slight adjustments occurred in Nansha District, Conghua District, and Zengcheng District under the coordination priority scheme, and minor changes were observed in Panyu District and Zengcheng District under the matching priority scheme, indicating stable overall spatial distribution patterns. These results demonstrate that the weight settings used are appropriate and that the research conclusions exhibit strong robustness.

**Table 7:** Sensitivity analysis of supply–demand matching results under different weighting schemes.

Index	Reference standard	Equal-weight scheme	Coordinated priority solution	Matching priority scheme
Coordination weight (a)	0.6	0.5	0.7	0.4
Matching weight (b)	0.4	0.5	0.3	0.6
Pearson correlation coefficient (compared with benchmark scheme)	—	0.9968	0.9976	0.9854
Number of administrative regions with changes in ranking	—	0	3	2
Change in supply and demand adaptation level	—	无	无	无

All Pearson correlation coefficients passed the significance test (*p* < 0.001).

## 5 Conclusion and discussion

### 5.1 Conclusion

This study takes the 11 administrative districts of Guangzhou as its research scope and comprehensively applies GIS spatial analysis, network-based accessibility evaluation, and supply–demand adaptability modeling to examine the spatial distribution, accessibility, and coordination between supply and demand of public older adults care services. The main conclusions are as follows:

Spatial clustering of supply and demand. Both older adults care service demand and supply in Guangzhou exhibit pronounced spatial clustering but differ significantly in their spatial distribution patterns. Demand is mainly concentrated in the traditional central districts—Yuexiu, Liwan, and Haizhu—characterized by high older adults population density, advanced aging levels, and heavy care burdens. In contrast, the supply of older adults care services presents a pattern of central concentration and peripheral diffusion. Community-based older adults care facilities, meal-assistance centers, medical-service sites, and public service institutions cluster within the central urban area, while older adults-care bed resources are more prevalent in outer districts such as Baiyun, Huangpu, and Huadu.

Spatial distribution characteristics of facilities. The spatial layout of public older adults care facilities demonstrates strong clustering features. Nearest-neighbor analysis confirms significant spatial clustering for all facility types; kernel-density results highlight hot spots concentrated in and around the central urban districts; and standard-deviation ellipses reveal that the general spatial orientation extends outward from the core to peripheral areas, forming an urban structure centered on the main city core.

Accessibility of public older adults care services. Overall accessibility in Guangzhou is relatively high, with most areas reachable within 15 min on foot, indicating broad spatial coverage of facilities. However, noticeable regional disparities remain: Yuexiu, Liwan, Haizhu, and Tianhe Districts exhibit superior accessibility, while northern Conghua and northeastern Zengcheng Districts show significantly lower accessibility and certain service gaps. Accessibility generally declines gradually from the central area toward the city’s periphery.

The supply–demand adaptability of public older adults care services in Guangzhou shows significant spatial differentiation characteristics. The supply and demand adaptation level of central urban areas such as Yuexiu District, Liwan District, and Haizhu District is relatively high, manifested in the coordinated development of older adults care service demand and resource supply; Tianhe District and Baiyun District are in a relatively compatible state; However, the adaptation level of Huangpu District, Panyu District, Huadu District, Nansha District, Conghua District, and Zengcheng District is relatively low, and some areas have problems of insufficient supply or low resource allocation efficiency. From the perspective of spatial pattern, the overall distribution characteristics of supply–demand adaptability are high in the center and low in the periphery.

### 5.2 Development suggestions

Based on the research findings, this study offers the following recommendations for optimizing the allocation of public older adults care resources in Guangzhou and promoting balanced development between older adults care supply and demand:

The research findings indicate that the demand and supply of older adults care services in central urban areas are highly concentrated, with a high degree of alignment between supply and demand. The central urban area should shift from resource allocation advantages to improving service quality. For central urban areas with high supply–demand adaptation levels such as Yuexiu District, Liwan District, and Haizhu District, more attention should be paid to improving the quality of older adults care services and optimizing service functions while maintaining the existing advantages of older adults care service resources. On the one hand, the community older adults care, meal assistance services, and medical older adults care integration service system can be further improved to enhance the professionalism and refinement of older adults care services; On the other hand, we should combine the multi-level and diversified needs of the older adults population, promote the expansion of older adults care services towards the integration of health and older adults care, smart older adult care, etc., and improve the efficiency of resource utilization and service supply quality of older adults care services.

Regarding the research findings indicating that the accessibility of public older adults care service facilities in peripheral urban areas and their alignment with supply–demand needs remain relatively low. For areas with relatively low levels of supply and demand adaptation, such as Huangpu District, Panyu District, Huadu District, Nansha District, Conghua District, and Zengcheng District, the layout of older adults care service facilities should be reasonably optimized according to the trend of population aging and changes in demand for older adults care services, and the coverage capacity of older adult care service resources should be improved. Among them, Conghua District and Zengcheng District can combine the characteristics of large regional area and relatively dispersed population distribution to improve the older adults care service network at the street and community levels, and enhance the accessibility of older adult care service facilities; Nansha District can leverage the opportunity of urban new area construction to coordinate the layout of older adults care service facilities and public service resources; Huangpu District, Panyu District, and Huadu District should further enhance the supply capacity of older adults care services, promote the allocation of older adults care resources to match the size of the older adults population.

Given the significant spatial disparities in the alignment between older adults care service supply and demand across different regions, it is essential to establish a robust dynamic monitoring mechanism to enhance service adaptability. A comprehensive dynamic monitoring system should be developed for tracking older adults care demand and resource allocation. By leveraging demographic statistics, data on older adults care facilities, and survey results on senior population needs, regular assessments should be conducted on regional aging trends, service capacity, and supply–demand balance, ensuring that facility planning aligns with demographic development patterns. Additionally, coordinated development between older adults care services and sectors such as healthcare, community governance, and digital technologies must be strengthened to build a comprehensive, rationally distributed, and efficiently operated older adults care service system that serves both urban and rural areas, thereby continuously improving the fairness and sustainability of resource allocation.

### 5.3 Research limitations and future research directions

This study still has certain limitations: it mainly constructs an evaluation system for the supply and demand of older adults care services based on indicators such as the number of older adults care facilities, bed resources, and population aging. Although it can better reflect the overall situation of older adults care service resource allocation, it has not fully considered individual differences such as the health status, income level, and preference for older adults care services of the older adults population, making it difficult to fully reveal the heterogeneous characteristics of older adults care service demand. In addition, the supply side indicators are mainly constructed based on indicators such as the number of public older adults care service facilities, bed resources, and POI spatial distribution, focusing on reflecting the spatial supply capacity and spatial configuration characteristics of older adults care service resources. However, they have not yet included indicators such as the number of employees in older adults care institutions, service quality, bed occupancy rate, and utilization rate that can reflect the capacity and operational efficiency of older adults care services. Therefore, the characterization of the level of older adults care service supply still has certain limitations. Due to limitations in data acquisition conditions, this article mainly analyzes the spatial configuration and service accessibility of older adults care service facilities, and has not yet comprehensively evaluated the supply of public older adults care services based on the quality, satisfaction, and actual utilization of older adults care services. In addition, this article conducts analysis based on cross-sectional data from 2024, mainly reflecting the allocation of public older adults care service resources at the research time point. Its dynamic evolution characteristics have not yet been revealed, nor has it been analyzed in conjunction with actual travel behavior and older adults care service selection preferences of the older adults. Therefore, the characterization of the demand response mechanism for older adults care services still needs further improvement.

Future research can combine questionnaire surveys, mobile location data, and long-term tracking data to further obtain behavioral data such as the actual travel behavior and preference for older adults care services of the older adults. Combined with indicators such as operational data of older adults care institutions, service quality evaluation, staffing and bed utilization, a more comprehensive evaluation system for public older adults care service supply can be constructed. At the same time, improved two-step mobile search method (2SFCA), spatial econometric model and other methods can be combined to comprehensively consider facility supply capacity, service competition and distance attenuation effects, and conduct in-depth analysis of the efficiency of older adults care service resource allocation and its dynamic evolution mechanism, thereby providing more accurate decision-making basis for the high-quality development of older adults care services.

### 5.4 Discussion

This study employs GIS spatial analysis, pedestrian accessibility assessments, and supply–demand matching models to reveal a distinct hierarchical differentiation pattern in Guangzhou’s public older adult care facilities characterized by “central concentration with decreasing density in peripheral areas,” a spatial feature reasonably explained by empirical findings. From a supply–demand perspective, the older adults population is highly concentrated in central districts such as Yuexiu, Liwan, and Haizhu, where robust local care demands have driven dense deployment of community-based older adults care services, meal assistance programs, and basic senior-friendly facilities, creating a balanced state of high supply and adequacy in core zones. In contrast, peripheral urban areas exhibit lower aging rates and limited care demand, resulting in overall low facility density and weaker supply–demand alignment—where spatial disparities in population needs serve as the primary driver of this stratification. Structurally, Guangzhou’s older adults care services demonstrate clear typological variations: central districts primarily feature embedded community-based services tailored for daily convenience, achieving high supply–demand compatibility; peripheral areas concentrate on large-scale nursing home resources focused on long-term rehabilitation, which inadequately meet the lower local care demands, leading to structural supply–demand mismatches. Regarding accessibility, central districts boast compact, densely distributed care facilities with strong service coverage overlap, essentially achieving 15-min walk accessibility for quality services; whereas remote suburban areas like Conghua and Zengcheng suffer from extensive spatial coverage gaps and sparse facility distribution, creating service blind spots that compromise accessibility and support efficiency in peripheral regions. In summary, the spatial imbalance in Guangzhou’s older adults care services stems from the interplay between the spatial concentration of demand, the differentiated distribution of facility types, and regional geographical conditions.

In terms of studying commonalities, this article confirms that the core agglomeration and peripheral weak circle pattern of older adults care resources in mega cities is consistent with the conclusions of most domestic urban older adults care space research, and is a universal spatial law for high-density urban older adults care resource allocation. This further confirms the core constraint effect of population agglomeration on the spatial layout of older adults care facilities, and validates the rationality and universality of the results of this study. From the perspective of research refinement differences, most existing studies only focus on the overall spatial pattern or homogeneous supply–demand matching evaluation of older adults care facilities, without distinguishing between the layout differences of different older adults care facilities and the differences in population demand. This article clearly distinguishes the spatial layout differences between community convenient older adult care services and institutional bed resources, accurately identifying the structural supply–demand mismatch problem in the inner and outer circles of Guangzhou. Compared with traditional single perspective research, it can more finely depict the structural contradictions in the allocation of older adult care resources in mega cities. At the same time, this article adopts a layered demand weighting calculation method to conduct supply and demand evaluation, which is different from the traditional research method of assigning values to the homogeneous population of the entire region. It effectively avoids the evaluation bias caused by unified weights and can accurately capture the hidden supply and demand shortcomings in suburban areas, new and old urban areas. It provides a more practical empirical approach for the fine adaptation evaluation of older adult care services in mega cities.

## Data Availability

The original contributions presented in the study are included in the article/supplementary material, further inquiries can be directed to the corresponding author.
